# Supplementary material for: Leveraging Physiologically Based Modelling to Provide Insights on the Absorption of Paliperidone Extended-Release Formulation under Fed and Fasting Conditions
Source: Pharmaceutics. 2023 Feb 13;15(2):629. doi: 10.3390/pharmaceutics15020629 (PMC9959113; doi:10.3390/pharmaceutics15020629)
Supplement: Supplementary file 1 [file pharmaceutics-15-00629-s001.zip › pharmaceutics-2155509-supplementary.pdf]

pharmaceutics

Leveraging Physiologically Based Modelling to Provide Insights on the Absorption of Paliperidone Extended Release Formulation under Fed and Fasting Conditions

Supplementary Materials

Saima Subhani[1,2], Viera Lucakova[2], Chaejin Kim[1], Leyanis Rodriguez-Vera[1], Paula Muniz[3], Monica Rodriguez[3], Rodrigo Cristofoletti[1], Sandra van Os[4], Elena Suárez[5], Stephan Schmidt[1], Valvanera Vozmediano[1*]

[1] Center for Pharmacometrics and System Pharmacology at Lake Nona (Orlando), Department of Pharmaceutics, College of Pharmacy, University of Florida, Orlando, FL, USA (S.S.), (C.K.), (L.R.V.), (R.C.), (St.S.), (V.V.)
[2] Simulations Plus, Lancaster, CA, USA (S.S.),(V.L.)
[3] Model Informed Development. CTI Laboratories Spain. , Derio, Bizkaia, Spain (P.M.), (M.R.)
[4] Synthon BV, Nijmegen, The Netherlands (S.O.)
[5]Department of Pharmacology, Pharmacokinetic, Nanotechnology and Gene Therapy Group (PharmaNanoGene), School of Medicine and Nursing, University of the Basque Country UPV/EHU, Biocruces Health Research Institute, Bizkaia, Spain. (E.S.)

Funding: This research received no external funding.

Conflict of Interest: Sandra van Os has no conflicts of interest other than being an employee of Synthon BV. Viera Lukacova has no conflicts of interest other than being an employee of Simulations Plus. All other authors declared no conflicts of interest directly relevant to the content of this manuscript.

Corresponding Author

Valvanera Vozmediano, Ph. D
Center for Pharmacometrics and System Pharmacology
Department of Pharmaceutics, College of Pharmacy,
University of Florida,
6550 Sanger RD. Orlando, FL. 32827. US
Phone: +1 407 313 7052
Email: valva@cop.ufl.edu

Contents

List of Tables

S1 Input parameters used to build the PBAM model in GastroPlus version 9.8 -----------2

S2 Comparison of simulated versus observed average PK parameters and metrics of single simulation of the oral solution under fasted condition------------------------------------5

S3 Verification of the PBAM model for the 2 mg PAL oral solution with external data from the literature---6

List of Figures

S1 Predicted versus observed PAL plasma concentrations over time after the oral administration of 1 mg oral solution using the final model--------------------------------------5

S2 Parameters sensitivity analysis showing the effect of changes on different absorption-related parameters on a) the area under the curve (AUC), b) the fraction absorbed (Fa) and c) maximum plasma concentrations (Cmax)--7

S3 Simulation of the upper extreme scenario (i.e., maximum Weibull release fixed to 97% and colonic transit time fixed to 34 h) with the final model. Maximum exposure prediction with the model is not able to capture the subject with higher exposure) --8

S4 External validation of OROS PAL. Predicted compared to observed PAL. (A) CMAX values, (B) AUC0-inf values from all literature studies---9

Table S1. Input parameters used to build the PBAM model in GastroPlus version 9.8

Parameter	Value	Source
Solubility at pH 6.8	0.39 mg/mL	[1]
Pka	2.6, 8.2	[2]
SolFactor	302.4(Pka=2.6), 4.429 (Pka=8.2)	Fitted to literature reported solubility [1] vs pH curve
Log P	1.02	[3]
Mean precipitation time	900 sec	GastroPlus version 9.8
Diff. coefficient	0.63 cm^2/sec X 10^5	ADMET predictor 9.5
Drug particle density	1.2 g/ml	ADMET predictor 9.5
Caco-2 permeability (Basolateral to Apical)	30.9 cm/s X 10^{-5}	[4]
Caco-2 permeability (Apical to Basolateral)	1.62 cm/s X 10^{-5}	[4]
Caco-2 permeability (Geometric Mean)	2.24 cm/s X 10^{-5}	[4]

S1. Input parameters used to build the PBAM model in GastroPlus version 9.8 (Continuation)

Structure	.sdf file of 2D structure	[3]
Molecular weight	426.49 g/mol	ADMET predictor 9.5
Molecular Formula	$C_{23}H_{27}FN_4O_3$	ADMET predictor 9.5
C1 for fasted or fed model	0.06944	Default GastroPlus version 9.8 (Human Physiology Fasted/Fed)
C2 for fasted or fed model	0.43028	Default GastroPlus version 9.8 (Human Physiology Fasted/Fed)
C3 for fasted or fed model	0.6	Fitted from 0.12147
C4 for fasted or fed model	0.8	Fitted from 0.46632
Volume of fluid in small intestine (ACAT model)	7.5%	Adjusted from 40% [5]
Volume of fluid in colon (ACAT model)	2%	Adjusted from 10% [5]
Renal Clearance (L/h/Kg)	0.0428	[6]
FPE (%)	2.78	[6]
K12	0.36846	Estimated by 3- compartment fit
K21	0.5136	Estimated by 3- compartment fit
K13	0.01457	Estimated by 3- compartment fit
K31	0.02098	Estimated by 3- compartment fit

Table S2. Comparison of simulated versus observed average PK parameters and metrics of single simulation of the oral solution under fasted condition

Parameter	Simulated Values	Observed Values	%PE
Fa (%)	99.888	N.A	N.A
FDp (%)	99.886	N.A	N.A
F (%)	97.102	N.A	N.A
C_{max} (ng/mL)	8.9655	8.5153	5.29%
T_{max} (h)	1.32	1.6102	-18.02%
$AUC_{0\text{-}inf}$ (ng-h/mL)	182.34	182.65	-0.17%
$AUC_{0\text{-}t}$ (ng-h/mL)	178.21	182.65	-2.43%

Fa(%), FDp (%), F(%) represent fraction absorbed across the apical membrane of the enterocyte, fraction absorbed reaching the portal vein, fraction bioavailable respectively, expressed as percentage of dose ; N.A: Not available.

Table S3. Verification of the PBAM model for the 2 mg PAL oral solution with external data from the literature

PK metrics (units)	Reported values* Mean (%CV)	Simulated values Mean (%CV)	Prediction Error (%)
Cmax (ng/ml)	19.4 (34%)	17.5 (19.14)	9.79
Tmax (h)	1.2 (47%)	1.85 (31.42)	
AUC (0-96h) (ng.h/ml)	371 (36%)	321.82 (11.68)	17.74
AUC (0-inf) (ng.h/ml)	397 (36%)	305.19 (10.9)	18.94

* patent-WO2007050377A1

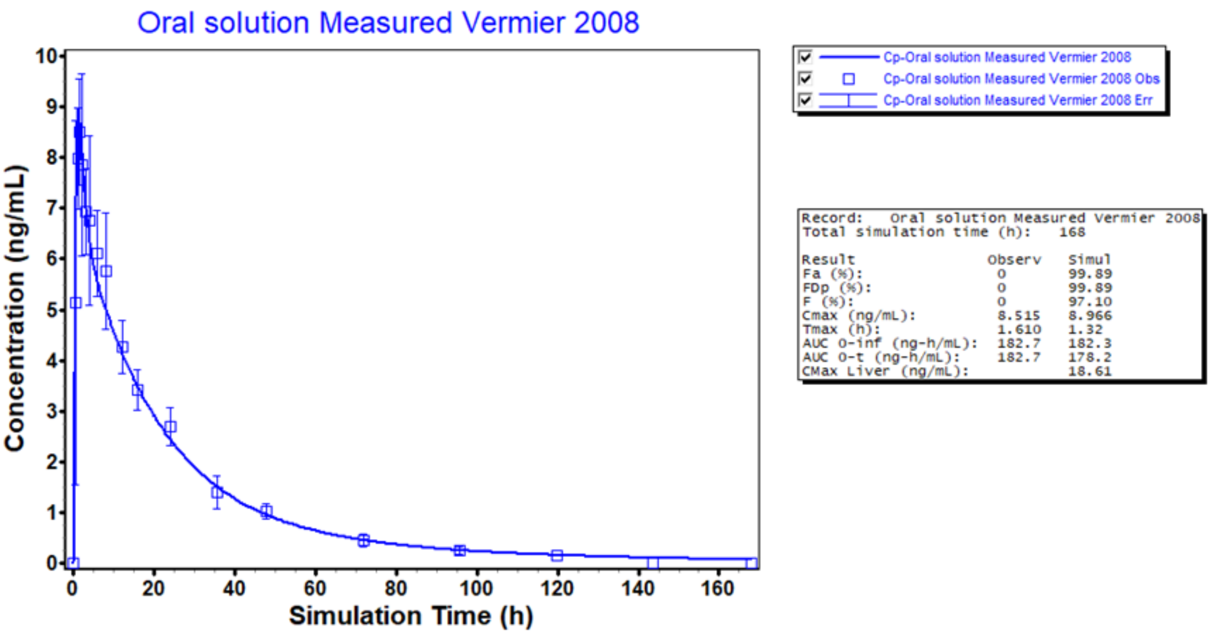

Figure S1. Predicted versus observed PAL plasma concentrations over time after the oral administration of 1 mg oral solution using the final model (blue solid line-predicted plasma concentration, blue square box with bars- observed mean plasma concentration in clinical fasted study ± standard error).

A

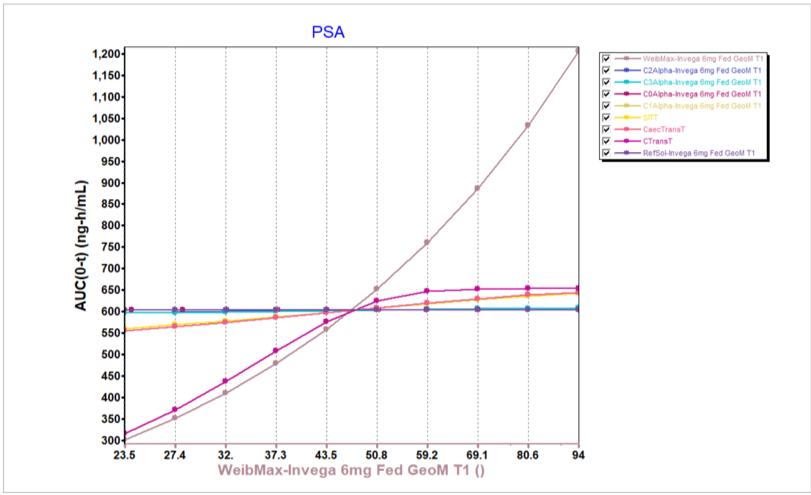

B

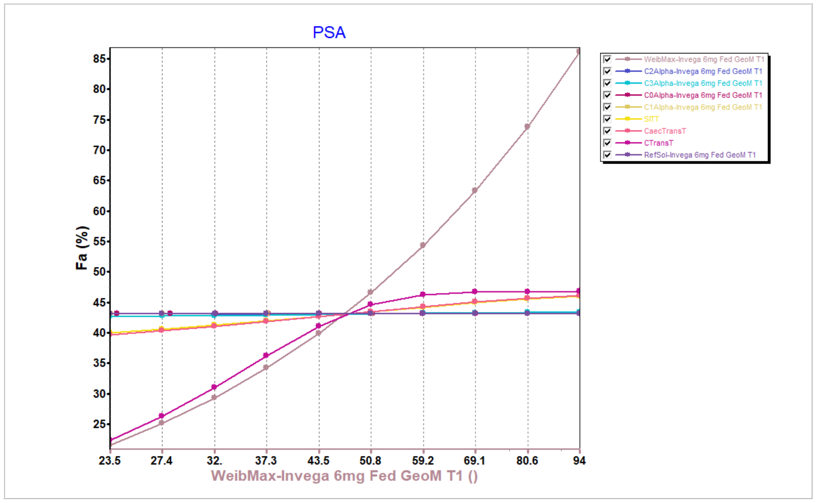

C

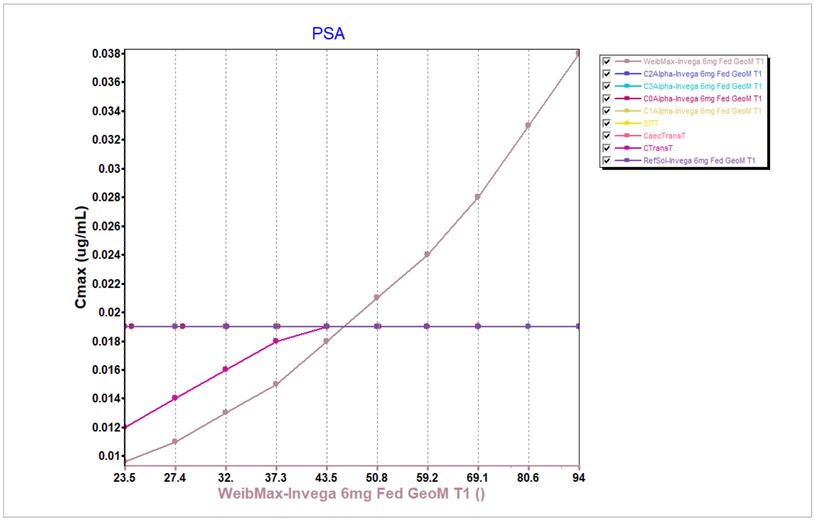

Figure S2. Parameters sensitivity analysis showing the effect of changes on different absorption-related parameters on A) the area under the curve (AUC), B) the fraction absorbed (Fa), and C) maximum plasma concentrations (Cmax).

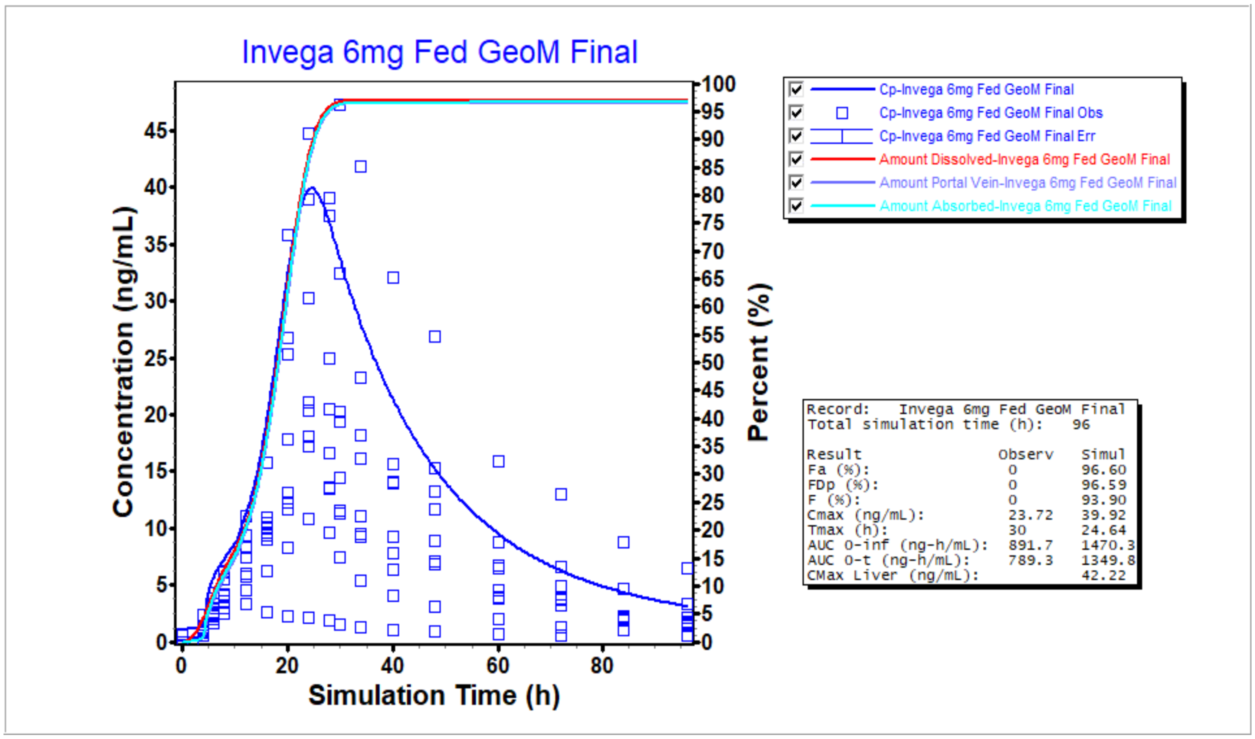

Figure S3. Simulation of the upper extreme scenario (i.e., maximum Weibull release fixed to 97% and colonic transit time fixed to 34 h) with the final model. Maximum exposure prediction with the model is not able to capture the subject with higher exposure.

A

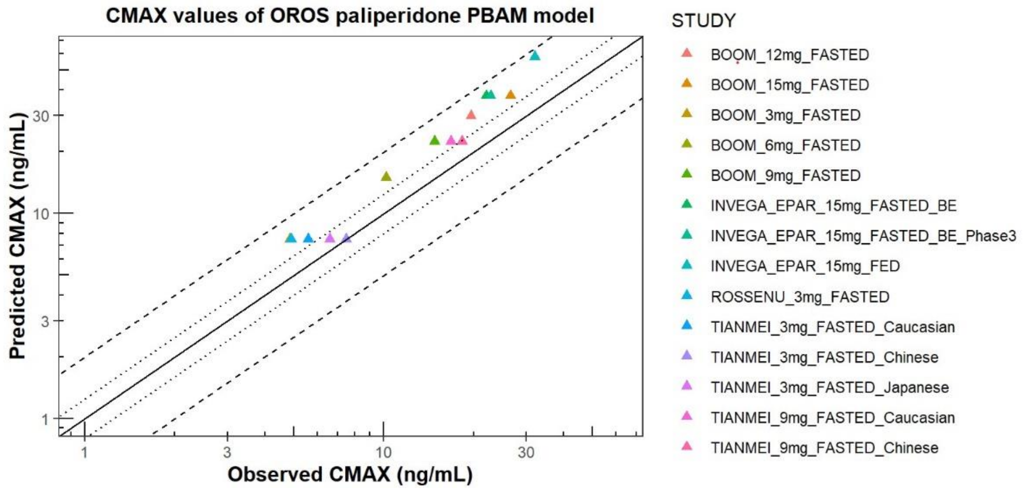

B

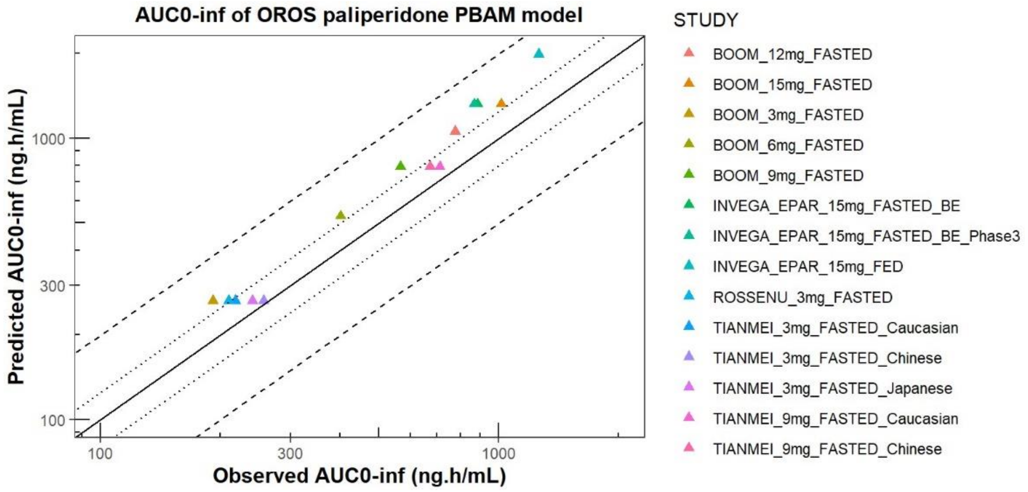

Figure S4. External validation of OROS PAL. Predicted compared to observed PAL. (A) CMAX values, (B) AUC0-inf values from all literature studies [7, 8, 9]. The line of identity is shown as a solid line; 1.25-fold deviation is shown as a dotted line; 2-fold deviation is shown as a dashed line. CMAX: maximum concentration; AUC: area under the curve.

References

1. Prabhakar, D.; Divya, A.; Prathyusha, R.; Kumar, K.S. Augmentation of Dissolution Profile of Poorly Soluble Paliperidone by employing Liquisolid Technology. Available online: https://sphinxsai.com/2014/PTVOL6/PT=39(710-719)AJ14.pdf (accessed on February 28th, 2022).
2. Product Monograph - Invega® paliperidone extended-release tablets (3 mg, 6 mg, 9 mg and 12 mg). Available online: https://pdf.hres.ca/dpd_pm/00043904.PDF (accessed on 10 Jan 2019).
3. PubChem Compound Summary for CID 115237, Paliperidone. Available online: https://pubchem.ncbi.nlm.nih.gov/compound/Paliperidone (accessed on 10 Jan 2019).
4. FDA. Paliperidone-New Drug Application: Amendment-Clinical Pharmacology and biopharmaceutics review (Application No.21-999). **2006**.
5. Sutton, S.C. Role of physiological intestinal water in oral absorption. *AAPS J* **2009**, *11*, 277-285, doi:10.1208/s12248-009-9087-2.
6. Vermeir, M.; Naessens, I.; Remmerie, B.; Mannens, G.; Hendrickx, J.; Sterkens, P.; Talluri, K.; Boom, S.; Eerdekens, M.; van Osselaer, N.; et al. Absorption, metabolism, and excretion of paliperidone, a new monoaminergic antagonist, in humans. *Drug Metab Dispos* **2008**, *36*, 769-779, doi:10.1124/dmd.107.018275.
7. Rossenu, S.; Crauwels, H.; Cleton, A., et al. Comparison of the pharmacokinetics of an oral immediate-release and extended-release formulation of paliperidone in healthy subjects. Poster presented at: The Annual Meeting and Exposition of the American Association of Pharmaceutical Sciences (AAPS) **2006**; October 29-November 2,; San Antonio, TX.
8. Tianmai S, Liang S, Yi L, et al. Single-dose pharmacokinetics of paliperidone extended-release tablets in healthy Chinese subjects. *Hum Psychopharmacol* **2010**, 25(5), 404-409.
9. Booms S, Talluri K, Janssens L, et al. Single- and multiple-dose pharmacokinetics and dose proportionality of the psychotropic agent paliperidone extended release. *J Clin Pharm* **2009**, 49,1318-1330
